# Climate and Dengue Transmission: Evidence and Implications

**DOI:** 10.1289/ehp.1306556

**Published:** 2013-09-20

**Authors:** Cory W. Morin, Andrew C. Comrie, Kacey Ernst

**Affiliations:** 1 School of Geography and Development, and; 2 The Mel and Enid Zuckerman College of Public Health, The University of Arizona, Tucson, Arizona, USA

## Abstract

Background: Climate influences dengue ecology by affecting vector dynamics, agent development, and mosquito/human interactions. Although these relationships are known, the impact climate change will have on transmission is unclear. Climate-driven statistical and process-based models are being used to refine our knowledge of these relationships and predict the effects of projected climate change on dengue fever occurrence, but results have been inconsistent.

Objective: We sought to identify major climatic influences on dengue virus ecology and to evaluate the ability of climate-based dengue models to describe associations between climate and dengue, simulate outbreaks, and project the impacts of climate change.

Methods: We reviewed the evidence for direct and indirect relationships between climate and dengue generated from laboratory studies, field studies, and statistical analyses of associations between vectors, dengue fever incidence, and climate conditions. We assessed the potential contribution of climate-driven, process-based dengue models and provide suggestions to improve their performance.

Results and Discussion: Relationships between climate variables and factors that influence dengue transmission are complex. A climate variable may increase dengue transmission potential through one aspect of the system while simultaneously decreasing transmission potential through another. This complexity may at least partly explain inconsistencies in statistical associations between dengue and climate. Process-based models can account for the complex dynamics but often omit important aspects of dengue ecology, notably virus development and host–species interactions.

Conclusion: Synthesizing and applying current knowledge of climatic effects on all aspects of dengue virus ecology will help direct future research and enable better projections of climate change effects on dengue incidence.

Citation: Morin CW, Comrie AC, Ernst KC. 2013. Climate and dengue transmission: evidence and implications. Environ Health Perspect 121:1264–1272; http://dx.doi.org/10.1289/ehp.1306556

## Introduction

Climate change is one of the most important environmental changes populations will face in the coming decades. Understanding how it may affect human health and disease is complex and requires a thorough understanding of links between present climate and disease (Epstein 2005). Links between climate and diseases with various modes of transmission (vector-, water-, food-, soil-, and airborne) have been identified (Colwell and Patz 1998; Epstein 2001), with the strongest associations being between climate and mosquito-borne diseases (Ebi et al. 2005; Rogers and Randolph 2000; Small et al. 2003). Although widely held as the world’s most important arbovirus, only one review of potential climate change impacts on dengue virus (DENV) transmission has been published with a focus on tools currently used to establish climate–disease associations (Thai and Anders 2011).

DENV is transmitted by *Aedes* genus mosquitoes, primarily *Aedes aegypti* and *Aedes albopictus*. Recent analysis indicates the that numbers of dengue fever (DF) cases may be as high as 400 million/year (Bhatt et al. 2013). Climate affects the DENV and vector populations both directly and indirectly (Gubler et al. 2001). Temperature influences vector development rates, mortality, and behavior (Christophers 1960; Rueda et al. 1990; Tun-Lin et al. 2000) and controls viral replication within the mosquito (Watts et al. 1987). Variability in precipitation influences habitat availability for *Ae. aegypti* and *Ae. albopictus* larvae and pupae. Temperature further interacts with rainfall as the chief regulator of evaporation, thereby also affecting the availability of water habitats. Indirectly, rainfall, temperature, and humidity influence land cover and land use, which can promote or impede the growth of vector populations. The incidence of DF has been associated with vegetation indices, tree cover, housing quality, and surrounding land cover (Troyo et al. 2009; Van Benthem et al. 2005). Climate change can also alter how humans interact with the land, altering its use and impacting mosquito population magnitude and species composition (Chang et al. 1997; Vanwambeke et al. 2007).

Although empirical relationships have been identified between climate conditions, DF, and DENV vectors, causal relationships have not been firmly established, thus limiting our ability to assess intervention strategies. In order to evaluate the potential impacts of climate change and better prepare mitigation strategies, we examined the strength of the evidence supporting the complex relationships among *Aedes* mosquitoes, DENV, and weather and climate. We also explored the relative utility of statistical and process-based models and their ability to identify key associations between climate and disease and to predict and simulate DENV transmission under projected climate change conditions.

## Methods

**Figure 1:**
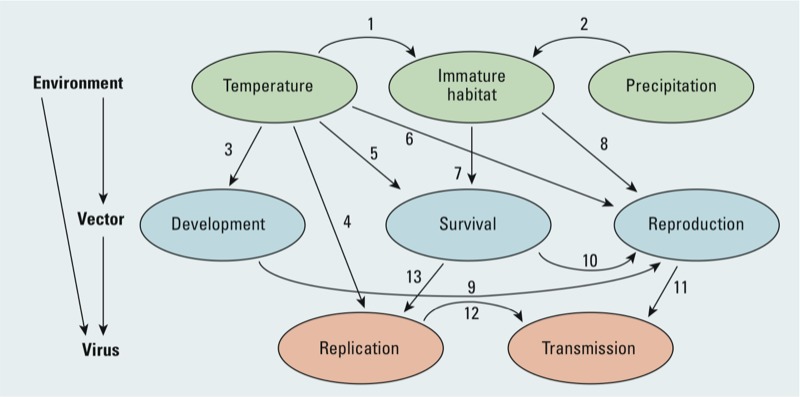
Diagram of biophysical influences on DENV ecology showing the interactions between climate variables, vectors, and the virus. Numbers identify relationships between variables. Habitat availability for mosquito larvae is influenced by temperature through evaporation and transpiration (*1*) and incoming precipitation (*2*). Temperature is a major regulator of mosquito development (*3*), viral replication within infected mosquitoes (*4*), mosquito survival (*5*), and the reproductive behavior of mosquitoes (*6*). Habitat availability is required for immature mosquito survival (*7*) and reproduction of adult mosquitoes (*8*). Faster mosquito development and increased survival will accelerate mosquito reproduction (*9* and *10*). Increased mosquito reproduction enhances the likelihood of transmission by increasing the number of blood feedings (*11*), whereas faster viral replication increases transmission by shortening the extrinsic incubation period (*12*). Last, increased survival of the adult mosquito increases the amount of viral replication (*13*).

We developed a framework of the hypothesized relationships between *Aedes* mosquitoes, DENV, and weather and climate (Figure 1). We reviewed evidence for each of the links illustrated in Figure 1 based on *a*) findings in published literature from laboratory and field studies conducted on the vector and virus, *b*) results of predictive models driven by weather and climate data, and *c*) investigations of DENV transmission in relation to climate. We assessed the relative strengths and limitations of empirical and process-based models and their use of established climate–transmission relationships. Last, we provide recommendations for future research to enhance model performance and improve our ability to forecast and prepare for the effects of climate change on DF.

## Results

*Temperature and virus replication and transmission*. Temperature is a key component in the ecology of DENV as seen from its numerous interactions with other components of the disease system (Figure 1). Most directly, ambient temperature increases are associated with a faster rate of viral replication within the vector and with a shorter extrinsic incubation period (EIP; the time required for DENV to become transmissible to another host after initial infection of a mosquito). There are four major serotypes of DENV: DENV-1, DENV-2, DENV-3, and DENV-4. Rohani et al. (2009) demonstrated that for both DENV-1 and DENV-4 the time between feeding and virus detection in the salivary glands of *Ae. aegypti* mosquitoes decreased from 9 days at 26°C and 28°C to 5 days at 30°C. Watts et al. (1987) more directly demonstrated that the EIP for DENV-2 virus in *Ae. aegypti* mosquitoes is temperature dependent by allowing infected mosquitoes to feed on monkeys. They reported that the EIP was as short as 7 days at temperatures of 32–35°C, and ≥ 12 days at 30°C, whereas no virus transmission occurred at 26°C within the 25-day period of the study (Watts et al. 1987). In addition, the EIP was dependent on the density of the virus in the ingested blood, such that higher densities were associated with shorter EIPs (Watts et al. 1987). Using censored Bayesian time-to-event models to analyze data collected from many studies, Chan and Johansson (2012) estimated the average EIP to be 15 days at 25°C, and 6.5 days at 30°C. Rohani et al. (2009) estimated shorter incubation periods (5–9 days) but they defined the end of the EIP as the time when the virus was detected in the mosquito, whereas Watts et al. (1987) defined it as the time when the mosquito transmitted the virus. Because temperature varies throughout the day in nature, Lambrechts et al. (2011) explored the susceptibility of *Ae. aegypti* to DENV infections under different diurnal temperature ranges (DTRs). They found that with the same mean temperature, mosquitoes exposed to a greater DTR were less likely to become infected than those exposed to a smaller DTR; however, the EIP was unchanged (Lambrechts et al. 2011). Evidence suggests that even small increases in temperatures and narrower DTRs may facilitate DENV transmission by decreasing the EIP or by increasing the susceptibility of mosquitoes to infection.

*Temperature and vector ecology*. Mosquitoes of the genus *Aedes,* primarily *Ae. aegypti*, are the vectors for DENV; therefore, the ecology of the virus is intrinsically tied to the ecology of these mosquitoes. Temperature can exert considerable influence on mosquito population dynamics (Scott et al. 2000b). Egg and immature mosquito development, ovarian development, and survival at all stages of the mosquito life cycle are governed in part by temperature (Christophers 1960). In the laboratory setting, Rueda et al. (1990) found that immature *Ae. aegypti* development rates generally increased with incubation temperatures to 34°C and then slowed. Survival through all developmental phases peaked at approximately 90% (27°C) with cooler temperatures being especially detrimental to survival (Rueda et al. 1990). Tun-Lin et al. (2000) reported that *Ae. aegypti* egg, larvae, and pupae development rates increased at higher incubation temperatures and ceased at < 8.3°C. Their estimated survival rates were also similar to those reported by Rueda et al. (1990), with the ideal range for survival through all phases of development (88–93%) occurring between 20–30°C (Tun-Lin et al. 2000). The laboratory studies discussed above yielded consistent results with little variation between trials. In the field portion of their study, Tun-Lin et al. (2000) found that development rates accelerated in warmer water, but development was often slower and more variable in field trials than in their laboratory trials at comparative temperatures.

Adult mosquito survival is important because only mosquitoes that live beyond the EIP can act as potential vectors. The first blood meal is generally taken 3 days post-eclosion (i.e., 3 days after emerging as an adult); therefore, assuming an EIP of 7–12 days (based on Watts et al. 1987), a minimum of 10–15 days is required for a newly emerged mosquito to become infectious. Mark–release–recapture studies have estimated that adult daily survival rates are between 86% and 91% (Muir and Kay 1998; Rebollar-Téllez et al. 1995). Although these studies did not examine climatic influences on survivability, Christophers (1960) has provided evidence of increased mortality with exposure to prolonged extreme heat (> 40°C) and cold (< 0°C) in a laboratory setting. Using the ranges of adult survival rates and the estimated minimum age to complete the EIP indicated above, 10.4% (86% daily survival with a 15-day EIP) to 38.9% (91% daily survival with a 10-day EIP) of mosquitoes will survive long enough to complete the EIP and become infectious to humans, assuming they are infected with DENV when they take their first blood meal. Larger DTRs result in a shorter mosquito life span than smaller DTRs around the same mean temperature; however, larger DTRs may concurrently shorten the duration of the EIP, demonstrating the complex nature of these relationships (Lambrechts et al. 2011). Carrington et al. (2013) recently reported that less thermal energy is required for pupation when temperatures fluctuate around a low mean value than when the temperature is constant at that value, whereas more thermal energy is required when temperatures fluctuate around a higher mean value than when the temperature is constant around that value. These results indicate that population models that assume the required thermal energy for pupation is constant could be over- or underestimating development times, leading to inaccurate simulations.

The female mosquito’s reproductive cycle is also governed by ambient temperature. At < 20°C, fertilization decreases (Christophers 1960). de Garin et al. (2000) established that increased minimum temperatures resulted in accelerated oviposition cycles and egg laying. Female *Ae. aegypti* require a blood meal for ovarian development, and feeding behavior is also influenced by temperature. Feeding activity is limited or ceases at temperatures < 15°C (Christophers 1960) and can also be limited at temperatures > 36°C (CW Morin, unpublished data). Multiplicity of feeding, that is, the taking of blood meals from multiple hosts during a single gonotrophic cycle (blood feeding, ovarian development, and egg laying), has been associated with higher levels of DENV transmission (Scott and Takken 2012). Scott et al. (2000a) found that higher temperatures were associated with higher incidences of multiple blood feedings in Thailand but not in Puerto Rico. Female size was also negatively correlated with temperature, and smaller females exhibited increased multiplicity of feeding in Thailand (Scott et al. 2000a).

The effect of temperature on the ability of the mosquito to reproduce has consequences for population dynamics and range limits. There is evidence of selection of breeding containers based on temperature and sun exposure. Barrera et al. (2006) found that *Ae. aegypti* preferred shaded containers and cooler water temperatures for egg laying in Puerto Rico. However, Wong et al. (2011) found that containers with more sun exposure were more likely to be inhabited in Iquitos, Peru. This may be because obtaining optimal water temperature for the mosquito required heating from direct sun exposure in one location and protection from the sun in another. In both cases, however, water temperature was important for mosquito reproduction. Barrera et al. (2006) also found the presence of trees to be associated with *Ae. aegypti* pupal productivity; they suggested that although dense vegetation may promote growth by contributing organic material to the habitat, it can also influence water temperature and evaporation by creating shade. This highlights how land-cover and land-use characteristics can influence microclimates and, consequently, mosquito populations. Using a statistical population model with known temperature limits and ecological parameters of the mosquito, Otero et al. (2006) calculated the limits of *Ae. aegypti* habitat range to be along the 15°C average annual temperature isotherm. An examination of the spatial distribution of adult *Ae. aegypti* with remotely sensed land use and land cover indicated that *Ae. aegypti* were more likely to be present in areas with structures and medium height trees than in areas with bare earth (Landau and van Leeuwen 2012).

**Figure 2:**
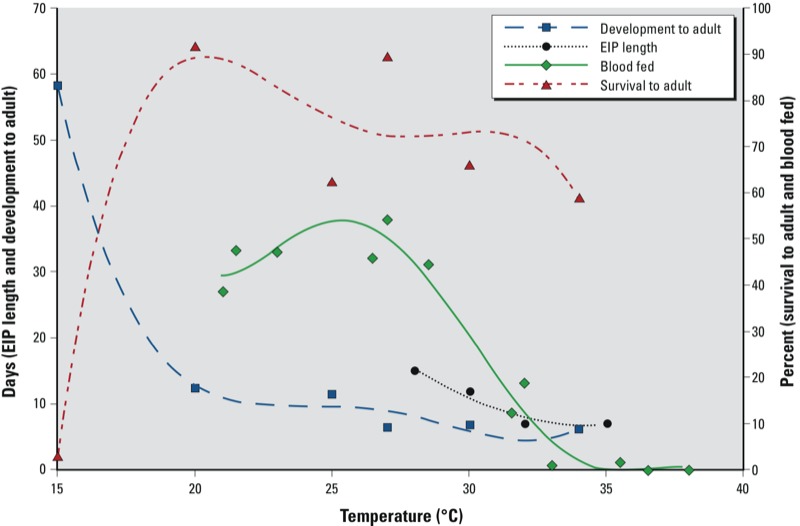
Effects of temperature on variables associated with DENV transmission. Days required for immature *Ae. aegypti* development to adult (Rueda et al. 1990), length of DENV-2 EIP (Watts et al. 1987), percent of *Ae. aegypti* mosquitoes that completed a blood meal within 30 min after a blood source was made available (Morin CW, unpublished data), and percent of hatched *Ae. aegypti* larvae surviving to adulthood (Rueda et al. 1990).

Although there are similarities in the thermal characteristics of many of the variables associated with vector and viral development, the critical limiting values are not the same. Integrating all these variables together in one temperature-driven model can enable simulation of very complex dynamics. Figure 2 shows the varying effects of temperature on many of the variables discussed above. There is often little correlation in the individual responses of these variables to temperature. This indicates that although increasing temperatures may accelerate parts of the viral transmission cycle, other components may become limited by high temperatures. Thus, the overall effect of warming on DENV ecology will be context dependent.

*Precipitation and vector ecology*. Whereas temperature has a direct biophysical influence on viral replication and on vector development and survival, precipitation provides essential habitat for the aquatic stages of the mosquito life cycle. Containers, common in urban environments, are often an important habitat (Southwood et al. 1972). Hoeck et al. (2003) studied vector abundances related to climate in Tucson, Arizona, an urban area inhabited by *Ae. aegypti* and in close proximity to DENV-endemic areas, but where DF does not occur. Not surprisingly, increased collections of *Ae. aegypti* eggs and adults coincided with the monsoon rains. In neighborhoods near San Juan, Puerto Rico, Barrera et al. (2011) found that higher precipitation was associated with increased *Ae. aegypti* populations, and that man-made containers were the most important pupae habitat for producing adult mosquitoes. Intense rainfall, however, may wash out breeding sites and thus have a negative effect on vector populations.

Precipitation is often required to create and maintain breeding sites and has a strong influence on vector distributions. Kolivras (2010) estimated intraannual climate-induced range changes of *Ae. albopictus* in Hawaii using a geographic information system (GIS), climate data, and the known habitat of the mosquito. They concluded that mosquito ranges expand during La Niña conditions (generally wetter) and decrease during El Niño conditions (generally drier). This could increase future risk of DF given projected changes of El Niño Southern Oscillation (ENSO) cycles (Kolivras 2010). Drier conditions, however, can indirectly expand a vector’s range. Kearney et al. (2009) used a biophysical model in conjunction with an evolutionary response component to project alterations in the range of *Ae. aegypti* in Australia due to climate change and concluded that habitat for the mosquito will likely expand as individuals increase household water storage in response to a drier climate. Drier conditions could also cause selection pressure towards greater egg resistance to desiccation (Kearney et al. 2009). Beebe et al. (2009) posited that installing domestic water reservoirs to combat drying from warmer temperatures and decreased precipitation actually provides additional breeding grounds for *Ae. aegypti* mosquitoes, whose range is predicted to expand with increasing temperature. Australia’s risk of DF comes not only from the direct effects of climate change on mosquito population density, but also from the adaptive measures people take to mitigate its effects.

*Climate, habitat, and vector ecology*. Precipitation and temperature work interdependently: Increased temperatures accelerate evaporation rates and limit standing water as a potential habitat source for immature mosquitoes, although the eggs are resistant to desiccation over extended time periods (Christophers 1960) and thus climate effects on immature vector survival are a complex balance between precipitation and evaporation. Higher rates of precipitation combined with higher temperatures also result in increased humidity. Higher humidity is associated with increased *Ae. aegypti* feeding activity, survival, and egg development (Christophers 1960). For instance, Nagao et al. (2003) reported that daily minimum temperature and an increase in precipitation from the previous month were both associated with increased larval abundances.

Within water habitats, competition for space and nutrients is a key determinant of population levels. As the water in containers evaporates, the density of immature mosquitoes may increase, enhancing competition and deterring potential egg laying. Barbosa et al. (1972) raised immature *Ae. aegypti* at various densities in laboratory conditions and found that higher densities resulted in slower development, greater mortality, and a lower body mass. Field studies have supported these findings (Seawright et al. 1977; Walsh et al. 2011). Southwood et al. (1972) demonstrated that mortality was density dependent for *Ae. aegypti* between the egg and second instar larval development stage. Similarly, Dye (1982) found that when competition existed among larvae, the younger instars suffered the greatest delay in development. At the greatest extreme, complete evaporation will result in complete mortality of larvae and pupae.

Much of the density dependence exhibited in these studies is the result of nutritional stress in the containers. This suggests that precipitation exerts less influence on density-dependent mortality than nutritional levels within the habitat. Keirans and Fay (1968) subjected laboratory-reared *Ae. aegypti* to varying levels of food rations and concluded that oviposition and pupation were delayed under food stress. Moore and Whitacre (1972) reported that nutritional levels regulate production of growth retardant factor (GRF), which can limit population growth. However, a conflicting study by Dye (1984) showed that the production of GRF is strain dependent, and it probably plays only a small role in population control. Still other studies have provided evidence that shows nutrition to be important for *Ae. aegypti* larval development and survival. Tun-Lin et al. (2000) discovered that containers with more organic matter resulted in larger mosquitoes, quicker development, and higher survival rates. Similarly, Barrera et al. (2006) studied larvae-inhabited containers in Puerto Rico and their results indicate the existence of food competition in most containers, although containers with a larger water volume showed reduced competition-related effects (such as retarded development and increased mortality). This may be explained by larger water bodies being less affected by evaporation, resulting in lower larvae/pupae population densities. Barrera et al. (2006) also showed that the body mass of individual mosquitoes was decreased among mosquitoes in more crowded containers. As noted previously by Scott et al. (2000a), smaller body size is associated with increased transmission potential because of the increased multiplicity of feeding in smaller mosquitoes compared with larger mosquitoes.

In addition, Zahiri and Rau (1998) reported that female preferences for oviposition sites were affected by larval density. Specifically, female *Ae. aegypti* were more attracted to sites with higher larval densities up to a threshold level, after which they were repelled (Zahiri and Rau 1998). These results indicate that the effects of precipitation and evaporation on available water sources can regulate the size, population, and behavior of DENV vectors.

Climate factors may also provoke competition between species of mosquitoes. *Ae. aegypti* and *Ae. albopictus* are two DENV vectors that often have overlapping habitat distributions. Juliano et al. (2002) studied egg mortality rates of these two species in varying laboratory environments and reported that although *Ae. aegypti* eggs thrived across a wide range of humidity and temperature combinations, *Ae. albopictus* eggs experienced high mortality at conditions at < 95% humidity when temperatures were > 22°C. In their southern Florida cemetery survey of mosquito presence vs. absence, Juliano et al. (2002) found a significantly lower *Ae. albopictus* presence after the dry season than during the wet season, whereas the *Ae. aegypti* presence was consistent.

*Vector population and DF incidence*. Although often monitored to assess DENV transmission risk, vector abundances are not always associated with DF incidence. In a study in São Paulo, Brazil, Dibo et al. (2008) reported that the average number of female mosquitoes was the best predictor of DF cases in humans and that numbers of female mosquitoes and eggs, and the presence of female mosquitoes were all positively correlated with DF cases (Dibo et al. 2008). However, studies by Chadee et al. (2007) and Wu et al. (2007), using the Breteau index (number of containers inhabited by immature *Ae. aegypti* per 100 houses inspected), did not find statistically significant relationships between mosquito indices and DF cases, suggesting that the utility of surveying vector populations to evaluate DF risk may vary. Several factors may be responsible for this lack of association. Chadee et al. (2007) posited that the development of herd immunity may explain the decrease in the incidence of DF observed over their 3-year study period despite a consistently high mosquito index. Wu et al. (2007) found temperature to be a significant predictor of DF incidence, and suggested that this relationship may exist because DENV replication within the mosquito is regulated by temperature and, therefore, so is the length of the EIP. It is possible that a given temperature may be sufficient to support vector populations but inadequate to support viral replication, or that once a certain threshold of mosquito density is met, a further increase in density will have only a minimal impact on DENV transmission.

*Climate and disease occurrence*. Numerous methods of statistical analysis have been used to estimate associations between climate variables and DF incidence, including cross correlations; Poisson, logistic, and multivariate regression; autoregression; and wavelet analysis (Table 1). Many have been successful (e.g., Arcari et al. 2007; Lu et al. 2009) in identifying climate–DENV relationships and creating predictive models of DF incidence based on climate associations. Variables that predict the intensity and timing of outbreaks include minimum, maximum, and mean temperature as well as relative humidity and wind velocity, whereas the seasonal timing of epidemics is predicted by precipitation. Often these variables are predictive at specific time lags.

**Table 1:** Studies identifying relationships between climate variables and DF cases.

Source	Location	Study type/model	Identified climate predictors/associations
Amarakoon et al. 2008	Caribbean	Time-series analysis, correlation	ENSO, temperature
Arcari et al. 2007	Indonesia	Multivariate regression, correlation	Temperature, rainfall
Barrera et al. 2011	Puerto Rico	Longitudinal study	Rainfall
Brunkard et al. 2008	Mexico	Time-series analysis, autoregressive model	Temperature, rainfall, sea surface temperature
Cazelles et al. 2005	Thailand	Wavelet analysis	ENSO
Chadee et al. 2007	Trinidad	Correlation	Precipitation
Chen et al. 2010	Thailand	Correlation, Poisson regression	Minimum temperature, rainfall, relative humidity
Chowell et al. 2011	Peru	Time-series analysis, spatial analysis	Mean temperature
Chowell and Sanchez 2006	Mexico	Correlation, multiple linear regression	Maximum temperature, evaporation, precipitation
Colón-González et al. 2011	Mexico	Multiple linear regression	Minimum temperature, ENSO
Descloux et al. 2012	Australia	Multivariate nonlinear model	Temperature, relative humidity, precipitation
Fuller et al. 2009	Costa Rica	Statistical model	ENSO
Gharbi et al. 2011	Guadeloupe	Seasonal autoregressive integrated moving average model	Relative humidity, mean temperature, minimum temperature
Hii et al. 2009	Singapore	Time-series analysis, Poisson regression	Mean temperature, precipitation
Hsieh and Chen 2009	Taiwan	Correlation, multiphase Richards model	Temperature, rainfall
Hurtado-Diaz et al. 2007	Mexico	Time-series analysis, autoregressive model	Sea surface temperature, minimum temperature, rainfall
Johansson et al. 2009a	Mexico, Puerto Rico, Thailand	Wavelet analysis	ENSO, temperature, precipitation (not uniformly)
Johansson et al. 2009b	Puerto Rico	Regression model	Temperature, precipitation
Jury 2008	Puerto Rico	Multiple statistics	Temperature, precipitation
Keating 2001	Puerto Rico	Multivariate linear regression	Temperature
Lu et al. 2009	China	Time-series analysis, Poisson regression	Minimum temperature, minimum humidity, wind velocity
Pinto et al. 2011	Singapore	Correlation, Poisson regression	Minimum and maximum temperature
Schreiber 2001	Puerto Rico	Multivariate regression	Temperature, energy, moisture variables
Su 2008	Philippines	Correlation, regression	Precipitation
Thai et al. 2010	Vietnam	Wavelet analysis	ENSO
Tipayamongkholgul et al. 2009	Thailand	Poisson autoregressive model	ENSO
Wu et al. 2007	Taiwan	Time-series analysis, autoregressive integrated moving average models	Monthly temperature variation, relative humidity
Wu et al. 2009	Taiwan	Spatial analysis, GIS	Temperature
Yu et al. 2011	Taiwan	Spatio­temporal analysis, stochastic Bayesian maximum entropy analysis	Multiple climate variables

The sign and strength of climate–DENV associations depend largely on local climate context. In a study by Yu et al. (2011) in southern Taiwan, mean and maximum temperature were negatively associated with DF cases, which seems counterintuitive until the generally high temperatures in this region [which can often reach levels that damage mosquitoes and thus limit virus transmission (> 30°C)] are considered. In contrast, in other regions low temperatures may be the limiting factor. Similarly, Pinto et al. (2011) did not find a high correlation between precipitation and DF incidence in Singapore because rain occurs there throughout the year, and thus it is not a significant limiting factor for mosquitoes. In Taiwan, Wu et al. (2009) found relative humidity to be negatively associated with DF incidence. They hypothesized that although mosquitoes generally survive longer in humid conditions, they may bite more when water-stressed, thus increasing DENV transmission at lower humidity (Wu et al. 2009). These studies highlight the multiple avenues through which climate variables can influence DENV transmission.

Because of their influence on weather patterns, ENSO indices and sea surface temperatures also have been analyzed in relation to DF incidence. Hurtado-Diaz et al. (2007) and Colón-González et al. (2011) have both found ENSO to be a good predictor of DF cases in Mexico. ENSO associations with DENV transmission have also been identified in the Caribbean (Amarakoon et al. 2008), Thailand (Cazelles et al. 2005; Tipayamongkholgul et al. 2009), Costa Rica (Fuller et al. 2009), Australia (Hu et al. 2010), and Vietnam (Thai et al. 2010). Studies in Noumea and Puerto Rico, however, have found little or only sporadic associations (Descloux et al. 2012; Johansson et al. 2009a). The inconsistent nature of these associations may reflect regional variation in the effects of ENSO on precipitation and temperature.

Unfortunately, because of data constraints, analyses are often performed using data aggregated over large spatial scales (e.g., country level) or long time periods (e.g., monthly or annually). Johansson et al. (2009a) found only weak associations between DF incidence, climate, and ENSO indices based on country-level analyses in Puerto Rico, Mexico, and Thailand. However, Johansson et al. (2009b) reported that several climate variables were associated with DF incidence when broken down by municipality in Puerto Rico. More important, the predictors and the strengths of their associations with DF varied among municipalities, highlighting the importance of local climate variation on DENV ecology. Other studies have also reported evidence of within-country or within-province variability in climate–dengue associations. In Indonesia, Arcari et al. (2007) found that variation in rainfall patterns among provinces was a large determinant of the strength and direction of associations between climate variables and DF incidence. Brunkard et al. (2008) reported that lag times for associations between climate variables and DF incidence varied among locations along the Texas–Mexico border, and Tipayamongkholgul et al. (2009) reported that the explanatory power of ENSO indices and climate on DF incidence varied by province in Thailand.

Analyses based on long time scales and broad geographic areas may fail to identify the influence of processes that occur over daily or weekly time periods and geography and climate features that may vary substantially at the country and sub-country level. For instance, Chowell et al. (2011) showed that timing of DF outbreaks in Peru varied considerably between the coastal, mountain, and jungle regions. In particular, because suitable climatic conditions for DENV transmission existed during most of the year in jungle regions, these regions were often the source of DF outbreaks in neighboring regions. Hsieh and Chen (2009) analyzed a multiwave outbreak of DF cases in Taiwan in 2007 and noted that after the initial wave of infection there were fewer infections in mid-August after two typhoons lowered temperatures, but the resulting moisture and remerging high temperatures resulted in a second wave of DF cases in early fall (Hsieh and Chen 2009). These patterns were evident because data were analyzed over weekly and daily time periods. Unfortunately, availability of data often forces researchers to scale up. For example, Barrera et al. (2011) attempted to perform a study at the census-block level in San Juan, Puerto Rico; however, because of the small numbers of DF cases, they had to analyze data at the municipality level instead.

The predictive power and robustness of predictive models would be improved with additional data over longer time periods. Gharbi et al. (2011), Hurtado-Diaz et al. (2007), and Tipayamongkholgul et al. (2009) created statistical predictive models that were trained on < 10 years of data and could only be tested over a 1-year period. This makes it difficult to assess whether the relationships they observed will hold in time. Schreiber (2001) created a statistical model for Puerto Rico using data from 1988 through 2005, and found that validation statistics were reduced considerably when data for a single year (1990) were removed. In Mexico, Colón-Gonzáles et al. (2011) noted an association between ENSO and DF incidence that was no longer evident when one large spike in DF cases was excluded from the analysis.

The reported incidence of DF is subject to substantial over- and underreporting, which may vary by geographic region and time. Changes in case definitions, health care availability, diagnostic capabilities, and subclinical cases all influence the number of cases reported. Descloux et al. (2012) avoided some of these issues by studying factors associated with time periods classified as epidemics or nonepidemics. It is also difficult to know where DENV transmission occurs, especially given the 4- to 10-day incubation period between infection and the onset of symptoms. Introduction of new DENV serotypes into a population can also be a confounding factor because it will alter immunity and symptoms in response to an incident infection. Finally, efforts to control DENV transmission may also confound associations between climate variables and the incidence of DF. Therefore, it is important to account for confounding by these and other factors before attempting to find associations with weather and climate.

Although the above mentioned studies suggest that climate strongly influences DENV transmission, some studies have found other factors to be of equal or greater importance. Wu et al. (2009) and Barrera et al. (2011) found the level of urbanization and number of artificial containers to be associated with vector populations and DF cases. Other studies have concluded that herd immunity, introduction of new or recycling of old viral serotypes, and changing demographics are partially or largely responsible for the variability in DENV transmission (Brunkard et al. 2008; Chadee et al. 2007; Keating 2001). Shang et al. (2010) found evidence that when climate conditions are favorable, imported cases of DF can initiate local DF epidemics in Taiwan, thus suggesting a way in which climate and human behavior may interact to influence viral transmission.

*Climate change and DF incidence*. The impact of climate change on DF has also been examined. The studies discussed above, concerning climate, vectors, and the virus, suggest that suitable climatic conditions are required for mosquito population development and subsequent infections. It follows, therefore, that changes in climate will alter the spatial and temporal dynamics of DENV ecology, potentially increasing vector ranges, lengthening the duration of vector activity, and increasing the mosquito’s infectious period by shortening the EIP. Conversely, increased temperatures in already warm locations may have negative effects on the range of virus transmission through decreased vector survival, reproduction, and immature habitat.

Jetten and Focks (1997) published one of the earliest papers connecting climate and future DENV transmission risk. They used a modified vectorial capacity equation to estimate the vector population required to maintain DENV transmission. The equation included climate dependent variables such as the EIP, daily mosquito mortality, mosquito size, and the length of the gonotrophic cycle. The Intergovernmental Panel on Climate Change–projected temperature increases of 2–4°C were applied to weekly-averaged data from weather stations in cities across the tropics. Jetten and Focks (1997) suggested that climate change would lead to an increase in the latitudinal and elevational extent of the disease, and a longer season of viral transmission.

Patz et al. (1998) used a similar vectorial capacity equation that was modified to estimate epidemic potential. The model used projected climate change data from a general circulation model (GCM) at 250 km × 250 km resolution to project future DF risk across the world, focusing on five climatologically contrasting cities. The model predicted large increases in the geographic extent of DF and a longer disease season, especially in temperate regions at the fringes of the virus’ range (Patz et al. 1998). Jetten and Focks (1997) and Patz et al. (1998) both provided a good framework for future research on climate and DF risk, but estimates should be updated based on current GCM projections with improved resolution that are based on standardized approaches.

Hales et al. (2002) conducted an early study using logistic regression to model disease range based on the statistical association of DF presence with water vapor and other climate variables. Based on GCM projections and human population demographics, the authors predicted that the magnitude and distribution of DF would increase and encompass a larger total population and percent of the population (Hales et al. 2002). Caution should be used when modeling DF incidence using statistical relationships with climate variables because the statistical model is trained with present climate data that may differ greatly from projected future climate regimes. The boundaries of statistical model predictions are limited to climate variable combinations that have already been experienced, and extrapolating outside those bounds may lead to inaccuracies.

Process-based models can be used to estimate the effects of climate change on DF incidence while avoiding some of the restrictions of statistical techniques. The container-inhabiting mosquito simulation model (CIMSiM) accomplishes this using a dynamic life-table model (Focks et al. 1993a, 1993b). CIMSiM estimates the combined effects of temperature, precipitation, container types, and predation on mosquito survival and development (Focks et al. 1993a, 1993b). This type of modeling has been applied using climate change scenarios to project changes in vector dynamics under future values of temperature and precipitation. Focks et al. (1995) paired CIMSiM with a DF simulation model (DENSiM) to simulate DENV transmission between mosquito and human populations. This work was the first to use a combined climate and epidemiological approach to study DENV ecology. The authors have conducted a preliminary validation of the model in Honduras but stress that, although initial results are promising, additional validation is needed. More recently, Schaeffer et al. (2008) used a matrix model driven by climate fluctuations to simulate the populations of two mosquito species of the genus *Aedes*. Using daily precipitation data, Schaeffer et al. (2008) were able to successfully replicate field populations of the mosquitoes over the first 2 years of the study period with relatively high accuracy. However, although the model replicated the general dynamics, it overestimated populations in the third year, possibly due to lower capture frequency or changing mortality rates. Hopp and Foley (2001) incorporated global average climate data that was linearly downscaled to 1° resolution into an *Ae. aegypti* population model. The authors reported that model-based estimates of mosquito density worldwide were in general agreement with the known mosquito range. In a follow-up study, Hopp and Foley (2003) used numeric modeling to simulate the response of *Ae. aegypti* mosquitoes to climate change. They correlated modeled mosquito populations with DF incidence and used this relationship to predict future DF incidence by modeling new mosquito populations using projected future climate data (Hopp and Foley 2003). Although these studies show progress in our understanding of DENV ecology, they are still limited by equating DF incidence with vector populations, which have been shown to be unrelated in some studies (Chadee et al. 2007; Wu et al. 2007). Newer models and studies should build upon this framework to represent finer scales and better-detailed processes.

Although process-based models can be used to consider the complex dynamics of DENV ecology, they possess important limitations as predictive tools. Detailed knowledge of the processes within the system is required to build dynamic models. This is not true of statistical models, which are capable of establishing relationships even if the mechanisms responsible for the relationships are not well understood. Indeed, associations identified based on statistical analyses can be used to inform the parameters used in process-based models, and process-based models can conversely identify field studies that might be useful. In addition, process-based models of well understood systems will not perform well without appropriate input data. Information on climate, land cover, and demographic data must be available and accurate to produce useful results. Lastly, certain relationships between components of the model may change over time in ways that are difficult to predict. This includes possible evolution of the virus and the vector, changes in the land use or land cover of an area, and the behavior of human populations. Evaluating model results can also be difficult. Factors that may confound the association between climate change and DF incidence include improvements in surveillance that increase the ascertainment of DF cases, making it difficult to determine if increased cases are related to improved surveillance or increases in regional or global temperatures. Therefore, although still an important and useful tool, the limitations of process-based models need to be considered, and despite the models described above having been successfully used to simulate past vector dynamics and DF epidemics, their utility for predicting future occurrences is untested.

Some studies suggest that climate change will not necessarily result in significant changes in the range and incidence of DF, especially in developed countries. Reiter et al. (2003) compared DF incidence levels on either side of the Texas–Mexico border and concluded that although mosquito levels were similar, the risk of DENV transmission was far lower in Texas than in Mexico because human–vector contact was reduced due to the prevalence of well-sealed air-conditioned buildings, less outdoor exposure, and socioeconomic factors (Reiter et al. 2003). Although not the focus of this review, the potential importance of socioeconomic factors should not be underestimated; clearly, these factors can have significant effects. In addition, we recognize that both mosquitoes and DENV may adapt to climate change in ways we cannot currently predict. However, the literature from other disease models suggests that the magnitude of these influences may vary by the intensity and types of climatic changes that occur in a given location. These changes may occur due to direct influences on the evolution of the vector and infectious agent but may also be related to changes in viral diversity associated with increasing or decreasing levels of DENV transmission (Artzy-Randrup et al. 2010; Liu et al. 2011; Randolph and Rogers 2002; Rottschaefer et al. 2011).

## Discussion

Previous studies have shown that climate exerts a critical influence on the spatial and temporal extent of DF. However, future studies need to better capture the effects of environmental factors on the ecology of DENV. Here we briefly discuss two examples of environmental influences that need to be resolved in future studies of climate and DENV transmission risk.

The assumption that a vector will always colonize new habitats if temperature tolerances allow is simplistic. Although mosquitoes transmitting disease are unlikely to be limited by host ranges (because they often feed primarily on humans), they may be limited by competition. For example in Florida, *Ae. aegypti* populations have been displaced spatially and/or temporally from some locations and replaced by *Ae. albopictus,* especially in rural settings (O’Meara et al. 1995), suggesting that *Ae. albopictus* may be a better competitor for larval habitat under certain conditions (Juliano 1998; Juliano et al. 2002; Leisnham and Juliano 2009; Lounibos et al. 2010; Murrell et al. 2011). Although both of these mosquitoes are DENV vectors, their competence is variable. *Ae. albopictus* tends to be a more generalist feeder, but it prefers human hosts for blood meals when available (Delatte et al. 2010; Kamgang et al. 2012; Ponlawat and Harrington 2005; Richards et al. 2006; Valerio et al. 2010). Studies also suggest that *Ae. albopictus* is less susceptible to DENV infection and dissemination to the salivary glands (Chen et al. 1993; Gratz 2004; Lambrechts et al. 2010; Moore et al. 2007; Vazeille et al. 2003). Physical barriers such as large water bodies, mountains, or deserts may also restrict species dispersion (Venkatesan and Rasgon 2010).

The effect of climate on the virus itself also has received little attention in the literature. Vector abundance indices are generally taken as the only measure of DENV transmission potential. Because climate exerts a major influence on mosquito population dynamics, studies focusing on climate–disease associations often choose to model vectors. However, virus dynamics within the mosquito must also be considered. Thomas et al. (2011), for example, used the temperature dependence of the EIP to project DENV transmission rates in Europe under various climate scenarios. Optimal temperatures for development of the vector are not necessarily the same as those for the virus (Figure 2). As a result, large vector populations may not be sufficient for transmitting DENV if viral replication is inhibited or if the lifespan of the mosquito is shorter than the EIP. Consequently, vector abundance/density may not always be appropriate proxy measurements of DENV transmission risk (Chadee et al. 2007; Wu et al. 2007). The virus and vector may also adapt to changes in climate as they occur slowly over time, and thus, changes in the relationship between climate and DENV transmission may also occur.

Future studies should focus on addressing these issues. DENV vector dispersal ability should be evaluated by inventorying the species currently inhabiting areas of concern and then assessing the likelihood of invasion by the new vector. Current mosquito population models can be made more sophisticated by including a viral component, as done by Focks et al. (1995). This entails calculating viral development within mosquitoes and ensuring that models are sensitive to adult survival and biting behavior in relation to temperature and humidity. The resulting models would be capable of estimating populations of infectious mosquitoes, and not simply total mosquito populations. Lastly, climate data can be incorporated into vectorial capacity models, as was done in earlier studies (Jetten and Focks 1997; Patz et al. 1998).

Many specific questions still need to be addressed. For instance: Do key processes identified in developing nations hold the same importance in developed nations? What is the best method of assessing climate effects on DENV transmission risk? Will human processes supersede environmental factors in dictating DF prevalence in some areas? And finally, can we enhance our understanding of DENV ecology by studying disease systems, and if so, how might we apply this knowledge to mitigate the effects of DENV on human populations?

## Conclusions

Although there has been much speculation on the connection between climate and DF occurrence, in the present review we have highlighted the need for research that produces more precise and stable results. Although climate variables strongly influence DENV and its vectors, caution must be taken when using only one element or connection to predict disease occurrence. Climate influences disease ecology at many levels, and the many nonlinearities and feedbacks present in the system create complex dynamics that are not easily modeled or understood. In addition, human factors, including behavior, immunity, and socioeconomic influences, also contribute to the complexity of these relations. Nonetheless, it may be possible to extract basic patterns and general predictions that could provide useful information for mitigating the effects of climate change on DF occurrences.

Capturing all aspects of the disease is a daunting task, but newer techniques may help overcome the difficulties. Process-based models that incorporate a more holistic view of the viral ecology should be implemented as new information on the topic is obtained and computing power increases. The use of interdisciplinary approaches will ensure that studies focus on the interactions between the components of the disease system, in addition to studying each component in isolation. A better understanding of the influences of climate on disease ecology is needed to improve projections of future disease risk, thus enabling better preparation and improved strategies to limit DENV transmission.

## References

[r1] Amarakoon D, Chen A, Rawlins S, Chadee DD, Taylor M, Stennett R (2008). Dengue epidemics in the Caribbean-temperature indices to gauge the potential for onset of dengue.. Mitig Adapt Strat Global Change.

[r2] Arcari P, Tapper N, Pfueller S (2007). Regional variability in relationships between climate and dengue/DHF in Indonesia.. Singapore J Trop Geo.

[r3] Artzy-Randrup Y Alonso D Pascual M 2010 Transmission intensity and drug resistence in malaria population dynamics: implications for climate change. PLoS One 5 e13588; 10.1371/journal.pone.0013588 21060886 PMC2965653

[r4] Barbosa P, Peters TM, Greenough NC (1972). Overcrowding of mosquito populations: response of larval *Aedes aegypti* to stress.. Environ Entomol.

[r5] Barrera R, Amador M, Clark GG (2006). Ecological factors influencing *Aedes aegypti* (Diptera: Culicidae) productivity in artificial containers in Salinas, Puerto Rico.. J Med Entomol.

[r6] Barrera R Amador M MacKay AJ 2011 Population dynamics of *Aedes aegypti* and dengue as influenced by weather and human behavior in San Juan, Puerto Rico. PLoS Negl Trop Dis 5 e1378; 10.1371/journal.pntd.0001378 22206021 PMC3243685

[r7] Beebe NW Cooper RD Mottram P Sweeney AW 2009 Australia’s dengue risk driven by human adaptation to climate change. PLoS Negl Trop Dis 3 e429; 10.1371/journal.pntd.0000429 19415109 PMC2671609

[r8] Bhatt S Gething PW Brady OJ Messina JP Farlow AW Moyes CL 2013 The global distribution and burden of dengue. Nature 496 7446 504 507; 10.1038/nature12060 23563266 PMC3651993

[r9] Brunkard JM, Cifuentes E, Rothenberg SJ (2008). Assessing the roles of temperature, precipitation, and ENSO in dengue re-emergence on the Texas-Mexico border region.. Salud Publica Mexico.

[r10] Carrington LB Armijos MV Lambrechts L Barker CM Scott TW 2013 Effects of fluctuating daily temperatures at critical thermal extremes on *Aedes aegypti* life-history traits. PLoS One 8 e58824; 10.1371/journal.pone.0058824 23520534 PMC3592833

[r11] Cazelles B Chavez M McMichael AJ Hales S 2005 Nonstationary influence of El Niño on the synchronous dengue epidemics in Thailand. PLoS Med 2 e106; 10.1371/journal.pmed.0020106 15839751 PMC1087219

[r12] Chadee DD, Shivnauth B, Rawlins SC, Chen AA (2007). Climate, mosquito indices and the epidemiology of dengue fever in Trinidad (2002–2004).. Ann Trop Med Parasitol.

[r13] Chan M Johansson MA 2012 The incubation periods of dengue viruses. PLoS One 7 e50972; 10.1371/journal.pone.0050972 23226436 PMC3511440

[r14] Chang MS, Hii J, Buttner P, Mansoor F (1997). Changes in abundance and behaviour of vector mosquitoes induced by land use during the development of an oil palm plantation in Sarawak.. Trans R Soc Trop Med Hyg.

[r15] Chen SC, Liao CM, Chio CP, Chou HH, You SH, Cheng YH (2010). Lagged temperature effect with mosquito transmission potential explains dengue variability in southern Taiwan: insights from a statistical analysis.. Sci Total Environ.

[r16] Chen W, Wei H, Hsu E, Chen E (1993). Vector competence of *Aedes albopictus* and *Ae. aegypti* (Diptera: Culicidae) to dengue 1 virus on Taiwan: development of the virus in orally and parenterally infected mosquitoes.. J Med Entomol.

[r17] Chowell G Cazelles B Broutin H Munayco CV 2011 The influence of geographic and climate factors on the timing of dengue epidemics in Peru, 1994–2008. BMC Infect Dis 11 164; 10.1186/1471-2334-11-164 21651779 PMC3121613

[r18] Chowell G, Sanchez F (2006). Climate-based descriptive models of dengue fever: the 2002 epidemic in Colima, Mexico.. J Environ Health.

[r19] Christophers SR. (1960). *Aedes aegypti* (L): The Yellow Fever Mosquito. Its Life History, Bionomics and Structure.

[r20] Colón-González FJ, Lake IR, Bentham G (2011). Climate variability and dengue fever in warm and humid Mexico.. Am J Trop Med Hyg.

[r21] Colwell RR, Patz JA. (1998). Climate, Infectious Disease and Health: an Interdisciplinary Perspective.

[r22] de Garin AB, Bejaran RA, Carbajo AE, de Casas SC, Schweigmann NJ (2000). Atmospheric control of *Aedes aegypti* populations in Buenos Aires (Argentina) and its variability.. Int J Biometeorol.

[r23] Delatte H, Desvars A, Bouétard A, Bord S, Gimonneau G, Vourc’h G (2010). Blood-feeding behavior of *Aedes albopictus*, a vector of chikungunya on La Réunion.. Vector Borne Zoonotic Dis.

[r24] Descloux E Mangeas M Menkes CE Lengaigne M Leroy A Tehei T 2012 Climate-based models for understanding and forecasting dengue epidemics. PLoS Negl Trop Dis 6 e1470; 10.1371/journal.pntd.0001470 22348154 PMC3279338

[r25] Dibo MR, Chierotti AP, Ferrari MS, Mendonca AL, Neto FC (2008). Study of the relationship between *Aedes* (*Stegomyia*) *aegypti* egg and adult densities, dengue fever and climate in Mirassol, state of São Paulo, Brazil.. Mem Inst Oswaldo Cruz.

[r26] Dye C (1982). Intraspecific competition amongst larval *Aedes aegypti*—food exploitation or chemical interference.. Ecol Entomol.

[r27] Dye C (1984). Competition amongst larval *Aedes aegypti*—the role of interference.. Ecol Entomol.

[r28] Ebi KL, Hartman J, Chan N, McConnell J, Schlesinger M, Weyant J (2005). Climate suitability for stable malaria transmission in Zimbabwe under different climate change scenarios.. Climatic Change.

[r29] Epstein PR (2001). Climate change and emerging infectious diseases.. Microbes Infect.

[r30] Epstein PR (2005). Climate change and human health.. New Engl J Med.

[r31] Focks DA, Daniels E, Haile DG, Keesling JE (1995). A simulation-model of the epidemiology of urban dengue fever—literature analysis, model development, preliminary validation, and samples of simulation results.. Am J Trop Med Hyg.

[r32] Focks DA, Haile DG, Daniels E, Mount GA (1993a). Dynamic life table model for *Aedes aegypti* (L.) (Diptera, Culicidae)–analysis of the literature and model development.. J Med Entomol.

[r33] Focks DA, Haile DG, Daniels E, Mount GA (1993b). Dynamic life table model for *Aedes aegypti* (L.) (Diptera, Culicidae)—simulation results and validation.. J Med Entomol.

[r34] Fuller DO, Troyo A, Beier JC (2009). El Niño Southern Oscillation and vegetation dynamics as predictors of dengue fever cases in Costa Rica.. Environ Res Lett.

[r35] Gharbi M Quenel P Gustave J Cassadou S La Ruche G Girdary L 2011 Time series analysis of dengue incidence in Guadeloupe, French West Indies: forecasting models using climate variables as predictors. BMC Infect Dis 11 166; 10.1186/1471-2334-11-166 21658238 PMC3128053

[r36] Gratz NG (2004). Critical review of the vector status of *Aedes albopictus*.. Med Vet Entomol.

[r37] Gubler DJ, Reiter P, Ebi KL, Yap W, Nasci R, Patz JA (2001). Climate variability and change in the United States: potential impacts on vector- and rodent-borne diseases.. Environ Health Persp.

[r38] Hales S, Dewet N, Maindonald J, Woodward A (2002). Potential effect of population and climate changes on global distribution of dengue fever: an empirical model.. Lancet.

[r39] Hii YL Rocklov J Ng N Tang CS Pang FY Sauerborn R 2009 Climate variability and increase in intensity and magnitude of dengue incidence in Singapore. Global Health Action 2; 10.3402/gha.v2i0.2036 PMC2799326 20052380

[r40] Hoeck PAE, Ramberg FB, Merrill SA, Moll C, Hagedorn HH (2003). Population and parity levels of *Aedes aegypti* collected in Tucson.. J Vector Ecol.

[r41] Hopp MJ, Foley JA (2001). Global-scale relationships between climate and the dengue fever vector, *Aedes aegypti*.. Climatic Change.

[r42] Hopp MJ, Foley JA (2003). Worldwide fluctuations in dengue fever cases related to climate variability.. Climate Res.

[r43] Hsieh YH, Chen CW (2009). Turning points, reproduction number, and impact of climatological events for multi-wave dengue outbreaks.. Trop Med Int Health.

[r44] Hu W, Clements A, Williams G, Tong S (2010). Dengue fever and El Niño/Southern Oscillation in Queensland, Australia: a time series predictive model.. Occup Environ Med.

[r45] Hurtado-Díaz M, Riojas-Rodríguez H, Rothenberg SJ, Gomez-Dantés H, Cifuentes E (2007). Short communication: impact of climate variability on the incidence of dengue in Mexico.. Trop Med Int Health.

[r46] Jetten TH, Focks DA (1997). Potential changes in the distribution of dengue transmission under climate warming.. Am J Trop Med Hyg.

[r47] Johansson MA Cummings DAT Glass GE 2009a Multiyear climate variability and dengue—El Niño Southern Oscillation, weather, and dengue incidence in Puerto Rico, Mexico, and Thailand: a longitudinal data analysis. PLoS Med 6 e1000168; 10.1371/journal.pmed.1000168 19918363 PMC2771282

[r48] Johansson MA Dominici F Glass GE 2009b Local and global effects of climate on dengue transmission in Puerto Rico. PLoS Negl Trop Dis 3 e382; 10.1371/journal.pntd.0000382 19221592 PMC2637540

[r49] Juliano SA (1998). Species introduction and replacement among mosquitoes: interspecific resource competition or apparent competition?. Ecology.

[r50] Juliano SA, O’Meara GF, Morrill JR, Cutwa MM (2002). Desiccation and thermal tolerance of eggs and the coexistence of competing mosquitoes.. Oecologia.

[r51] Jury MR (2008). Climate influence on dengue epidemics in Puerto Rico.. Int J Environ Health Res.

[r52] Kamgang B Nchoutpouen E Simard F Paupy C 2012 Notes on the blood-feeding behavior of *Aedes albopictus* (Diptera: Culicidae) in Cameroon. Parasit Vectors 5 57; 10.1186/1756-3305-5-57 22433236 PMC3342132

[r53] Kearney M, Porter WP, Williams C, Ritchie S, Hoffmann AA (2009). Integrating biophysical models and evolutionary theory to predict climatic impacts on species’ ranges: the dengue mosquito *Aedes aegypti* in Australia.. Funct Ecol.

[r54] Keating J (2001). An investigation into the cyclical incidence of dengue fever.. Soc Sci Med.

[r55] Keirans JE, Fay RW (1968). Effect of food and temperature on *Aedes aegypti* (L.) and *Aedes triseriatus* (Say) larval development.. Mosq News.

[r56] Kolivras KN (2010). Changes in dengue risk potential in Hawaii, USA, due to climate variability and change.. Climate Res.

[r57] Lambrechts L, Paaijmans KP, Fansiri T, Carrington LB, Kramer LD, Thomas MB (2011). Impact of daily temperature fluctuations on dengue virus transmission by *Aedes aegypti*.. Proc Natl Acad Sci USA.

[r58] Lambrechts L Scott TW Gubler DJ 2010 Consequences of the expanding global distribution of *Aedes albopictus* for dengue virus transmission. PLoS Negl Trop Dis 4 e646; 10.1371/journal.pntd.0000646 20520794 PMC2876112

[r59] Landau KI, van Leeuwen WJD (2012). Fine scale spatial urban land cover factors associated with adult mosquito abundance and risk in Tucson, Arizona.. J Vector Ecol.

[r60] Leisnham PT, Juliano SA (2009). Spatial and temporal patterns of coexistence between competing *Aedes* mosquitoes in urban Florida.. Oecologia.

[r61] Liu K, Tsujimoto H, Cha S, Agre P, Rasgon JL (2011). Aquaporin water channel AgAQP1 in the malaria vector mosquito *Anopheles gambiae* during blood feeding and humidity adaptation.. Proc Natl Acad Sci USA.

[r62] Lounibos LP, O’Meara GF, Juliano SA, Nishimura N, Escher RL, Reiskind MH (2010). Differential survivorship of invasive mosquito species in south Florida cemeteries: Do site-specific microclimates explain patterns of coexistence and exclusion?. Ann Entomol Soc Am.

[r63] Lu L Lin H Tian L Yang W Sun J Liu Q 2009 Time series analysis of dengue fever and weather in Guangzhou, China. BMC Public Health 9 395; 10.1186/1471-2458-9-395 19860867 PMC2771015

[r64] Moore CG, Whitacre DM (1972). Competition in mosquitos. 2. Production of *Aedes aegypti* larval growth retardant at various densities and nutrition levels.. Ann Entomol Soc Am.

[r65] Moore PR, Johnson PH, Smith GA, Ritchie SA, Van Den Hurk AF (2007). Infection and dissemination of dengue virus type 2 in *Aedes aegypti, Aedes albopictus*, and *Aedes scutellaris* from the Torres Strait, Australia.. J Am Mosq Control Assoc.

[r66] Muir LE, Kay BH (1998). *Aedes aegypti* survival and dispersal estimated by mark-release-recapture in northern Australia.. Am J Trop Med Hyg.

[r67] Murrell EG, Damal K, Lounibos LP, Juliano SA (2011). Distributions of competing container mosquitoes depend on detritus types, nutrient ratios, and food availability.. Ann Entomol Soc Am.

[r68] Nagao Y, Thavara U, Chitnumsup P, Tawatsin A, Chansang C, Campbell-Lendrum D (2003). Climatic and social risk factors for *Aedes* infestation in rural Thailand.. Trop Med Int Health.

[r69] O’Meara GF, Evans LF, Gettman AD, Cuda JP (1995). Spread of *Aedes albopictus* and decline of *Ae. aegypti* (Diptera, Culicidae) in Florida.. J Med Entomol.

[r70] Otero M, Solari HG, Schweigmann N (2006). A stochastic population dynamics model for *Aedes aegypti*: formulation and application to a city with temperate climate.. Bull Math Biol.

[r71] Patz JA, Martens WJM, Focks DA, Jetten TH (1998). Dengue fever epidemic potential as projected by general circulation models of global climate change.. Environ Health Perspect.

[r72] Pinto E, Coelho M, Oliver L, Massad E (2011). The influence of climate variables on dengue in Singapore.. Int J Environ Health Res.

[r73] Ponlawat A, Harrington LC (2005). Blood feeding patterns of *Aedes aegypti* and *Aedes albopictus* in Thailand.. J Med Entomol.

[r74] Randolph SE, Rogers DJ (2002). Remotely sensed correlates of phylogeny: tick-borne flaviviruses.. Exp Appl Acarol.

[r75] Rebollar-Téllez EA, Loroño-Pino MA, Rodríguez-Angulo EM, Farfán-Ale JA (1995). Blood-feeding frequency and life expectancy of *Aëdes aegypti* (Diptera: Culicidae) in an urban area of Merida city, state of Yucatan, Mexico.. Revista Biomédica.

[r76] Reiter P, Lathrop S, Bunning M, Biggerstaff B, Singer D, Tiwari T (2003). Texas lifestyle limits transmission of dengue virus.. Emerg Infect Dis.

[r77] Richards SL, Ponnusamy L, Unnasch TR, Hassan HK, Apperson CS (2006). Host-feeding patterns of *Aedes albopictus* (Diptera: Culicidae) in relation to availability of human and domestic animals in suburban landscapes of central North Carolina.. J Med Entomol.

[r78] Rogers DJ, Randolph SE (2000). The global spread of malaria in a future, warmer world.. Science.

[r79] Rohani A, Wong YC, Zamre I, Lee HL, Zurainee MN (2009). The effect of extrinsic incubation temperature on development of dengue serotype 2 and 4 viruses in *Aedes aegypti* (L.).. Southeast Asian J Trop Med Public Health.

[r80] Rottschaefer SM Riehle MM Coulibaly B Sacko M Niare O Morlais I 2011 Exceptional diversity, maintenance of polymorphism, and recent directional selection on the *APL1* malaria resistance genes of *Anopheles gambiae*. PLoS Biol 9 e1000600; 10.1371/journal.pbio.1000600 21408087 PMC3050937

[r81] Rueda LM, Patel KJ, Axtell RC, Stinner RE (1990). Temperature-dependent development and survival rates of *Culex quinquefasciatus* and *Aedes aegypti* (Diptera, Culicidae).. J Med Entomol.

[r82] Schaeffer B, Mondet B, Touzeau S (2008). Using a climate-dependent model to predict mosquito abundance: application to *Aedes* (*Stegomyia*) *africanus* and *Aedes* (*Diceromyia*) *furcifer* (Diptera: Culicidae).. Infect Genet Evol.

[r83] Schreiber KV (2001). An investigation of relationships between climate and dengue using a water budgeting technique.. Int J Biometeorol.

[r84] Scott TW, Amerasinghe PH, Morrison AC, Lorenz LH, Clark GG, Strickman D (2000a). Longitudinal studies of *Aedes aegypti* (Diptera: Culicidae) in Thailand and Puerto Rico: blood feeding frequency.. J Med Entomol.

[r85] Scott TW, Morrison AC, Lorenz LH, Clark GG, Strickman D, Kittayapong P (2000b). Longitudinal studies of *Aedes aegypti* (Diptera: Culicidae) in Thailand and Puerto Rico: population dynamics.. J Med Entomol.

[r86] Scott TW, Takken W (2012). Feeding strategies of anthropophilic mosquitoes result in increased risk of pathogen transmission.. Trends Parasitol.

[r87] Seawright JA, Dame DA, Weidhaas DE (1977). Field survival and ovipositional characteristics of *Aedes aegypti* and their relation to population-dynamics and control.. Mosq News.

[r88] Shang CS Fang CT Liu CM Wen TH Tsai KH King CC 2010 The role of imported cases and favorable meteorological conditions in the onset of dengue epidemics. PLoS Negl Trop Dis 4 e775; 10.1371/journal.pntd.0000775 20689820 PMC2914757

[r89] Small J, Goetz SJ, Hay SI (2003). Climatic suitability for malaria transmission in Africa, 1911–1995.. Proc Natl Acad Sci USA.

[r90] Southwood TRE, Murdie G, Yasuno M, Tonn RJ, Reader PM (1972). Studies of the life budget of *Aedes aegypti* in Wat Samphaya, Bangkok, Thailand.. Bull World Health Organ.

[r91] Su GLS (2008). Correlation of climatic factors and dengue incidence in Metro Manila, Philippines.. Ambio.

[r92] Thai KT, Anders KL (2011). The role of climate variability and change in the transmission dynamics and geographic distribution of dengue.. Exp Biol Med.

[r93] Thai KT Cazelles B Nguyen NV Vo LT Boni MF Farrar J 2010 Dengue dynamics in Binh Thuan province, southern Vietnam: periodicity, synchronicity and climate variability. PLoS Negl Trop Dis 4 e747; 10.1371/journal.pntd.0000747 20644621 PMC2903474

[r94] Thomas SM, Fischer D, Fleischmann S, Bittner T, Beierkuhnlein C (2011). Risk assessment of dengue virus amplification in Europe based on spatio-temporal high resolution climate change projections.. Erdkunde.

[r95] Tipayamongkholgul M Fang CT Klinchan S Liu CM King CC 2009 Effects of the El Niño-Southern Oscillation on dengue epidemics in Thailand, 1996–2005. BMC Public Health 9 422; 10.1186/1471-2458-9-422 19930557 PMC2785791

[r96] Troyo A, Fuller DO, Calderon-Arguedas O, Solano ME, Beier JC (2009). Urban structure and dengue fever in Puntarenas, Costa Rica.. Singap J Trop Geogr.

[r97] Tun-Lin W, Burkot TR, Kay BH (2000). Effects of temperature and larval diet on development rates and survival of the dengue vector *Aedes aegypti* in north Queensland, Australia.. Med Vet Entomol.

[r98] Valerio L, Marini F, Bongiorno G, Facchinelli L, Pombi M, Caputo B (2010). Host-feeding patterns of *Aedes albopictus* (Diptera: Culicidae) in urban and rural contexts within Rome province, Italy.. Vector Borne Zoonotic Dis.

[r99] Van Benthem BHB, Vanwambeke SO, Khantikul N, Burghoorn-Maas C, Panart K, Oskam L (2005). Spatial patterns of and risk factors for seropositivity for dengue infection.. Am J Trop Med Hyg.

[r100] Vanwambeke SO, Lambin EF, Eichhorn MP, Flasse SP, Harbach RE, Oskam L (2007). Impact of land-use change on dengue and malaria in northern Thailand.. Ecohealth.

[r101] Vazeille M, Rosen L, Mousson L, Failloux AB (2003). Low oral receptivity for dengue type 2 viruses of *Aedes albopictus* from Southeast Asia compared with that of *Aedes aegypti*.. Am J Trop Med Hyg.

[r102] Venkatesan M, Rasgon JL (2010). Population genetic data suggest a role for mosquito-mediated dispersal of West Nile virus across the western United States.. Mol Ecol.

[r103] Walsh RK, Facchinelli L, Ramsey JM, Bond JG, Gould F (2011). Assessing the impact of density dependence in field populations of *Aedes aegypti*.. J Vector Ecol.

[r104] Watts DM, Burke DS, Harrison BA, Whitmire RE, Nisalak A (1987). Effect of temperature on the vector efficiency of *Aedes aegypti* for dengue 2 virus.. Am J Trop Med Hyg.

[r105] Wong J Stoddard ST Astete H Morrison AC Scott TW 2011 Oviposition site selection by the dengue vector *Aedes aegypti* and its implications for dengue control. PLoS Negl Trop Dis 5 e1015; 10.1371/journal.pntd.0001015 21532736 PMC3075222

[r106] Wu PC, Guo HR, Lung SC, Lin CY, Su HJ (2007). Weather as an effective predictor for occurrence of dengue fever in Taiwan.. Acta Trop.

[r107] Wu PC, Lay JG, Guo HR, Lin CY, Lung SC, Su HJ (2009). Higher temperature and urbanization affect the spatial patterns of dengue fever transmission in subtropical Taiwan.. Sci Total Environ.

[r108] Yu HL, Yang SJ, Yen HJ, Christakos G (2011). A spatio-temporal climate-based model of early dengue fever warning in southern Taiwan.. Stoch Environ Res Risk Assess.

[r109] Zahiri N, Rau ME (1998). Oviposition attraction and repellency of *Aedes aegypti* (Diptera: Culicidae) to waters from conspecific larvae subjected to crowding, confinement, starvation, or infection.. J Med Entomol.

